# Prognostic significance of cytokeratin 19 expression in pancreatic neuroendocrine tumor: A meta-analysis

**DOI:** 10.1371/journal.pone.0187588

**Published:** 2017-11-14

**Authors:** Dong Cen, Jiang Chen, Zheyong Li, Jie Zhao, Xiujun Cai

**Affiliations:** Department of General Surgery, Institute of Minimally Invasive Surgery, Sir Run Run Shaw Hospital, College of Medicine, Zhejiang University, Hangzhou, Zhejiang, China; University of South Alabama Mitchell Cancer Institute, UNITED STATES

## Abstract

**Background:**

Pancreatic neuroendocrine tumor (PNET) comprises 1–2% of all pancreatic tumors, but its incidence is increasing. Although many studies have investigated the correlation between cytokeratin 19 (CK-19) and PNET, the prognostic significance of CK-19 expression in PNET is inconclusive.

**Methods:**

Eligible studies were retrieved from Pubmed, Elsevier, Embase, Cochrane Library and Web of Science databases. All relevant data were extracted to analyze the relationship between CK-19 and PNET. We utilized a fixed or random effects model to calculate the pooled odds ratio (OR) with 95% confidence intervals (CI).

**Results:**

Pooled data indicated CK-19 expression was significantly associated with poor 3- and 5-year overall survival (OS) for PNET, but not for 1-year overall survival. Additionally, positive CK-19 expression was correlated with large tumor size, advanced differentiation grade in World Health Organization-2010 (WHO-2010) and WHO-2004, vascular invasion, lymph node metastasis and liver metastasis.

**Conclusions:**

Positive CK-19 expression can be used as a predictor of poor prognosis of PNET.

## Introduction

Pancreatic neuroendocrine tumor (PNET) accounts for 1–2% of all pancreatic neoplasms [1]. In the United States, an age-adjusted annual incidence of PNET is 0.3 cases per 100,000 people, and the incidence is steadily increasing [2, 3]. According to the World Health Organization-2010 (WHO-2010) classification system for PNET, tumors are divided into well-differentiated (Grade 1 and Grade 2) and poorly differentiated neuroendocrine carcinomas (Grade 3) [4]. Although the 10-year overall survival of patients with low grade (Grade 1 and 2) reaches 60–70%, PNET may recur or metastasize. Median survival of Grade 3 is less than 2 years [5]. Moreover, it is difficult to predict the clinical behaviors of PNET because the clinicopathological features range from benign to malignant [6]. Therefore, the identification of new biomarkers for estimating PNET prognosis is urgently important.

Cytokeratin (CK), mainly expressed in epithelial cells and skin appendages, is the largest subgroup of intermediate filament proteins [7]. They participate in the formation of the cell skeleton and play an important role in the response to stress, cell signaling and apoptosis [8]. CK is classified into acidic protein type I (CK-9-CK-20) and basic protein type II (CK-1-CK-8) [7]. Cytokeratin-19 (CK-19) belongs to type I and is comprised of 399 amino acids with a molecular weight of 44 kilodaltons. It contains a 13 amino acid extension of the alpha-helical rod without the carboxyterminal, non-alpha-helical tail domain [9]. CK-19 is mainly expressed in ductal epithelial (bile and pancreatic duct, renal collecting ducts) and gastrointestinal epithelia [10]. In the pancreas, CK-19 is normally expressed in the exocrine ducts but not in the exocrine acinar and endocrine islet cells. During pancreatic morphogenesis, CK-19 positive duct-like pancreatic precursor cells develop into exocrine acinar and islet beta-cell without CK-19 expression [11].

CK-19 expression was detected in PNET. Many studies have been performed to estimate the impact of CK-19 expression on the prognostic significance of PNET, but findings remain controversial. Therefore we performed a meta-analysis to investigate whether and how CK-19 expression impacted PNET prognosis.

## Methods

### Study selection

A systematic primary web search was performed of the Pubmed, Elsevier, Embase, Cochrane Library and Web of Science databases for articles published from 1990 to December 31, 2016. We used the following terms: ((pancreatic endocrine tumor) OR (pancreatic endocrine neoplasm) OR (pancreatic neuroendocrine tumor) OR (pancreatic neuroendocrine neoplasm)) AND ((keratin 19) OR (Cytokeratin 19) OR (Cytokeratin-19)). All eligible articles were selected, and their reference lists were scrutinized for additional available studies.

### Criteria for inclusion and exclusion

All studies included in this meta-analysis fulfilled the following criteria: (1) PNET was confirmed by pathology and not restricted by age or ethnicity; (2) CK-19 expression was measured by immunohistochemistry (IHC); (3) clinical trials or reports were published in English; (4) relevant data was provided directly or could be calculated indirectly; (5) the study with the highest quality assessment was enrolled when trials were performed in the same patient samples. Abstracts, editorials, letters, expert opinions, conference records, book sections, reviews without original data, case reports or studies without control groups were excluded. Studies were also excluded if: (1) the articles were about animals or cell lines; (2) the outcomes or parameters of patients were not clearly reported; (3) articles were overlapping.

First, the title and abstract were screened to see whether they fulfilled the inclusion criteria. Second, the full text was further assessed after the initial screening. Finally, the eligibility of studies was verified by two reviewers (DC and JC).

### Data extraction and literature quality assessment

Two reviewers (DC and JC) extracted valid data independently from eligible studies, and any discrepancy was resolved by consensus. Relevant characteristics were: (1) first author’s name; (2) publication date; (3) number of patients included in this meta-analysis; (4) characteristics of the study population, such as age, gender and clinicopathological features; (5) PNET stage according to WHO-2010 and WHO-2004 classification; (6) methods for evaluating CK-19 expression; (7) manufacturers of antibody; (8) percentage of CK-19 expression; (9) whether overall survival data was provided (Table 1).

**Table 1:** Characteristics of studies included in the meta-analysis.

Study	Year	Countray	Number of Patients	Mean Age	Gender(M/F)	Level of Evidence	WHO grade(2004)	WHO grade(2010)	Clinicopathological Feature	Method	Clone Number of Antibody(Source)	Dilution	Increased CK-19 Expression	Definition Standard	Provided OS Data
**Son et al (14)**	2015	Korea	182	51.4±13.10	81/101	6	NR	G1, G2, G3	TS, LN, VI, PI	IHC	Cell Marque, Rocklin, CA, USA	1:100	97/182	>5%	YES
**Jovenel Cherenfant (15)**	2014	America	128	55 ± 14	71/57	6	NR	G1, G2, G3	NR	IHC	Biocare, Concord, CA	1:10	82/128	NR	NO
**Xu et al (6)**	2013	China	100	NR	NR	6	NR	G1, G2, G3	LN, LM	IHC	Dako, Glostrup, Denmark	1:300	70/100	>5%	YES
**Zhang et al (16)**	2011	America	97	53.9 (54; 22–82)	51/46	6	NR	NR	VI, PI	IHC	RCK108, DAKO, Carpinteria, CA	1:20	58/97	>5%	NO
**Jonkers et al (18)**	2007	Netherlands	50	NR	NR	4	B, UB, M	NR	TS	IHC	RCK108, MUbio products BV, Maastricht, The Netherlands	1:200	14/50	>5%	YES
**S La Rosa (17)**	2007	Italy	136	NR	NR	4	B, UB, WDEC, PDEC	NR	NR	IHC	RCK108, DAKO	1:100	30/136	>5%	YES
**Jonkers et al (19)**	2006	Netherlands	22	NR	NR	4	B, UB, M	NR	NR	IHC	RCK108, Mubio, Maastricht, the Netherlands	1:200	4/22	>10%	NO
**Ali et al (20)**	2006	Canada	56	49.8	26/30	6	NR	NR	TS, LN, LM	IHC	Novocastra, Newcastle, UK	1:500	33/56	>5%	NO
**Deshpande et al (21)**	2004	America	54	NR	NR	4	NR	NR	NR	IHC	Dako Co., Carpenteria,CA	1:10	28/54	NR	YES
**Gurevich et al (22)**	2003	Russia	29	47	9/20	5	NR	NR	TS, LN, VI, PI, LM	IHC	RCK108, Dako (Glostrop, Denmark)	1:100	12/31	>10%	NO

NR, not reported; B, benign; UB, uncertain behavior; M, malignant; WDEC, well-differentiated endocrine carcinomas; PDEC, poorly differentiated endocrine carcinomas; G1, grade 1; G2, grade 2; G3, grade 3; TS, tumor size; VI, vascular invasion; PI, perineural invasion; LN, lymph node metastasis; LM, liver metastasis; IHC, immunohistochemistry; OS, overall survival

Our two reviewers assessed the quality of each selected study using the Newcastle-Ottawa scale (NOS) [12]. The evaluation of the methodology included three aspects: selection, comparability, and outcome or exposure. Final scores ranged from 0 (the least eligible) to 9 (the most eligible). The study would be ruled out if the score was less than 3.

### Statistical analysis

The statistical analysis was performed by Review Manager (RevMan) software (version 5.3; Cochrane collaboration, http:ims.cochrane.org/revman/download) and STATA (version 12.0, Stata Corp. College Station, Texas). We pooled statistical variables contained in the original studies directly and obtained variables from available data indirectly or by reading the Kaplan-Meier survival curve according to the method by Parmar MK [13]. The Odd ratio (OR), together with 95% confidence interval (CI), was analyzed to estimate the relationship between CK-19 expression and the prognosis of PNET. A combined OR<1 suggested a worse survival rate, and for clinicopathological features, a combined OR>1 indicated a poor survival outcome. Heterogeneity among enrolled studies was checked with a Chi-square-based Q statistical test. And the I^2^ statistic, ranging from 0% to 100%, was also calculated to measure the inter-study heterogeneity. If a P<0.10 and/or I^2^>50%, indicating the presence of heterogeneity, a random-effects model was used. Otherwise, a fixed-effects model was chosen. The publication bias was evaluated by the funnel plots made by Egger’s test and Begg’s test. If the plots were asymmetrical, the stability of our meta-analysis results needed to be assessed using trim and fill analyses. P<0.05 in the Q statistical test was considered statistically significant.

## Results

### Selection of trials

**Fig 1:**
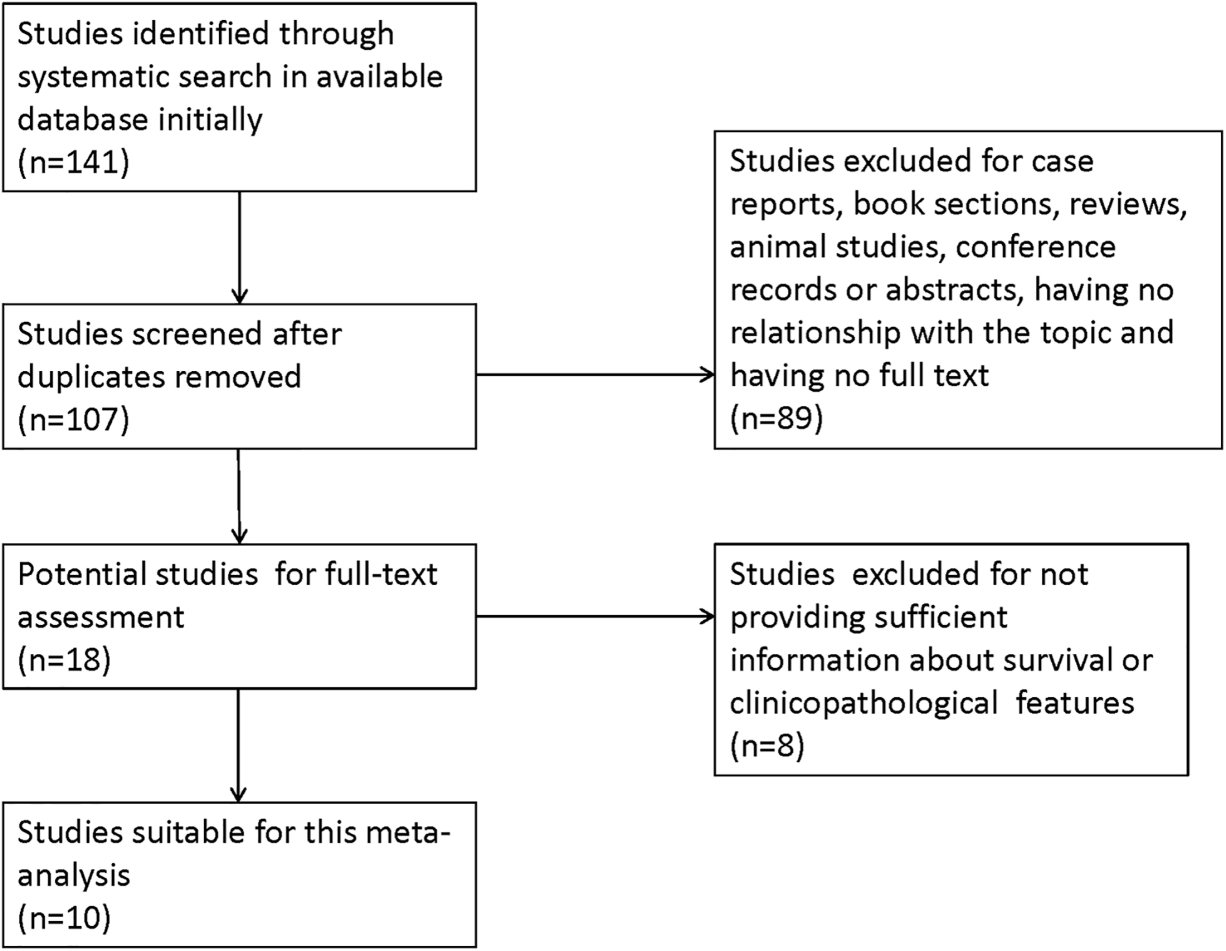
Flow chart of literature search strategies.

A total of 141 studies were retrieved based on the initial search criteria. 34 duplicate articles were excluded. Another 89 studies were excluded because they were case reports, book sections, reviews, animal studies, conference records or abstracts, or had no relationship with the topic or no full text. After reading the full text, we excluded 8 more studies because the information about survival or clinicopathological features was insufficient. At the end of the screening, 10 retrospective studies met inclusion criteria and were used in this meta-analysis [6, 14–22] (Fig 1).

### Study characteristics

The basic clinical characteristics of all enrolled studies are presented in Table 1. These studies were performed in Korea (1), China (1), Italy (1), Russia (1), Canada (1), the United States (3), and the Netherlands (2). All studies used IHC to detect CK-19 expression. Among 856 tissue samples, 428 showed positive CK-19 expression. According to the NOS evaluation, 5 studies scored 6, 1 study scored 5 and 4 studies scored 4.

### Meta-analysis of overall survival

**Fig 2:**
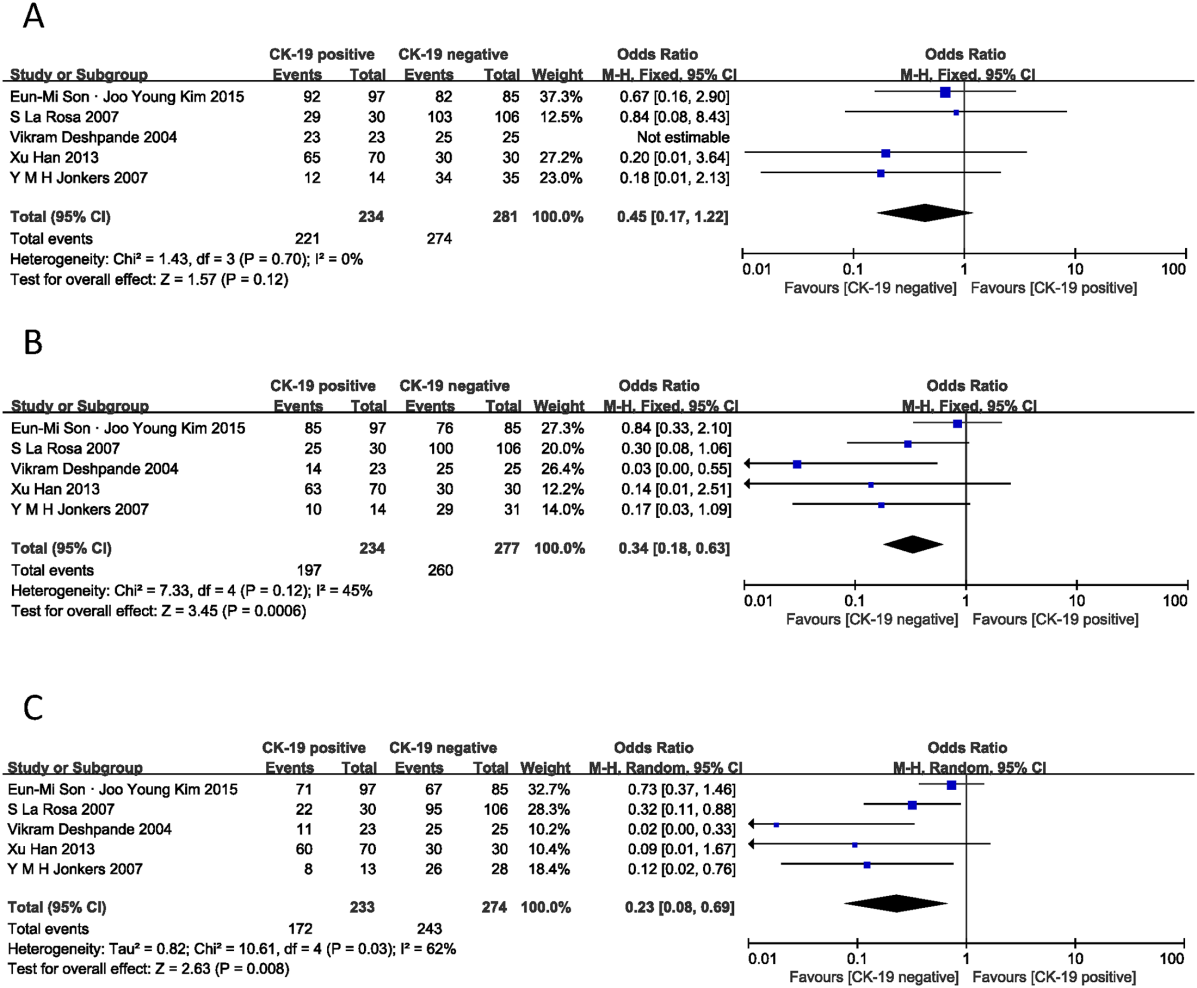
Forest plot displaying the results of the meta-analysis on overall survival of 1/3/5 year.

On the basis of the 5 studies [6, 14, 17, 18, 21], we investigated the correlation between CK-19 expression and PNET overall survival. As shown in Fig 2, the analysis was grouped into three phases: 1-year, 3-year and 5-year. The combined ORs were 0.45 (95% CI: 0.17–1.22, Z = 1.57, P = 0.12) for 1-year overall survival, 0.34 (95% CI: 0.18–0.63, Z = 3.45, P = 0.0006) for 3-year overall survival with no statistical heterogeneity (I^2^ = 0% and 45%). The pooled overall survival was 0.23 (95% CI: 0.08–0.69, Z = 2.63, P = 0.008) for 5-year overall survival with significant statistical heterogeneity (I^2^ = 62%). These values indicate that positive CK-19 was related to poor overall survival for PNET patients in both long and short phases, suggesting positive CK-19 expression is a prognostic indicator for PNET.

### Meta-analysis of clinicopathology

**Fig 3:**
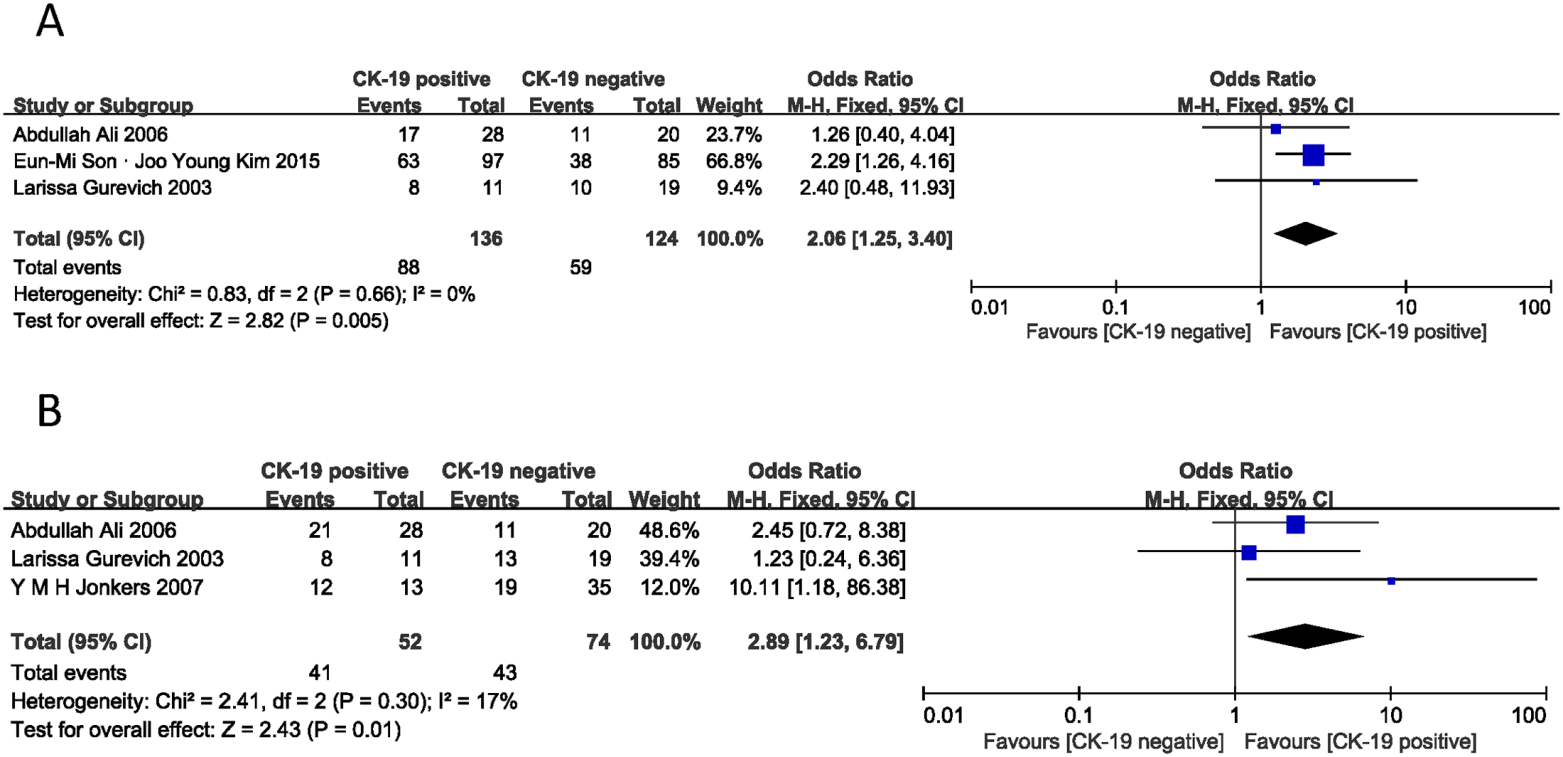
Forest plot displaying the results of the meta-analysis on tumor size.

Four studies assessed the correlation of CK-19 expression and tumor size [14, 18, 20, 22]. In one study, size equal to 2 centimeters (cm) belonged to small size group [14]. However, in another study, 2cm was classified in a large size group [18]. The remaining 2 studies described the tumor size and CK-19 expression of every tissue sample [20, 22]. Therefore, two analyses were performed according to whether 2 cm is included in large tumors or not (Fig 3). The combined ORs were 2.06 (n = 3 studies, 95% CI: 1.25–3.40, Z = 2.82, P = 0.005) and 2.89 (n = 3 studies, 95% CI: 1.23–6.79, Z = 2.43, P = 0.01) without statistical heterogeneity (I^2^ = 0% and 17%), indicating that CK-19 expression was associated with larger tumor size.

**Fig 4:**
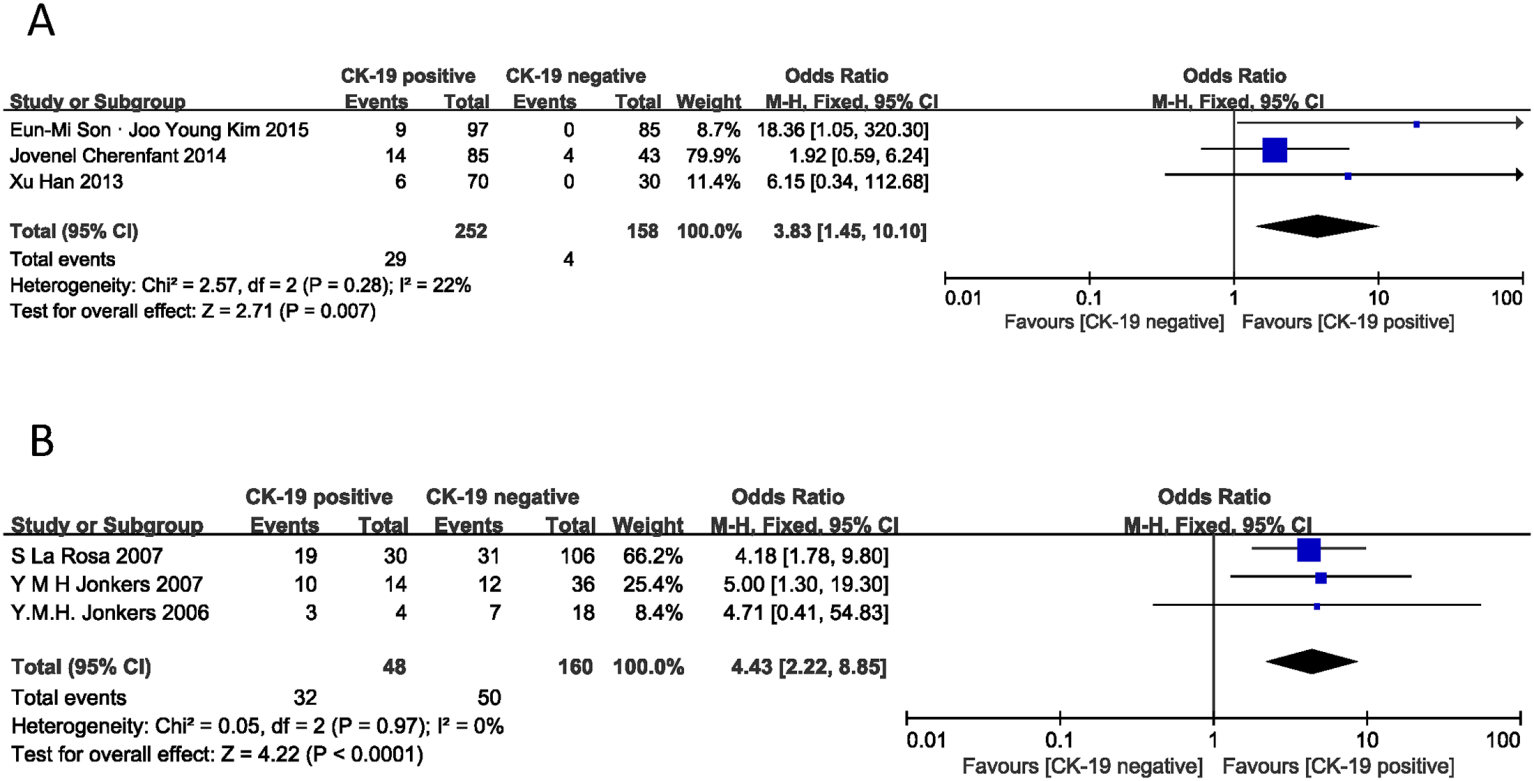
Forest plot displaying the results of the meta-analysis on differentiation grade of WHO-2010 and WHO-2004.

WHO-2010 and WHO-2004 are two classification systems for PNET. They both have clinical meaning for the differentiation grade of PNET. There were three studies investigating the effect of CK-19 expression on the WHO-2010 classification system [6, 14, 15]. The pooled analysis demonstrated that CK-19 expression had an impact on the advanced differentiation grade, Grade 3. The combined OR was 3.83 (95% CI: 1.45–10.10, Z = 2.71, I^2^ = 22%, P = 0.007) (Fig 4). Another 3 studies provided the data about CK-19 expression with the WHO-2004 classification system [17–19]. The pooled OR was 4.43 (95% CI: 2.22–8.85, Z = 4.22, I^2^ = 0%, P<0.0001) (Fig 4), suggesting that there was a significant relationship between positive CK-19 expression and malignant PNET.

**Fig 5:**
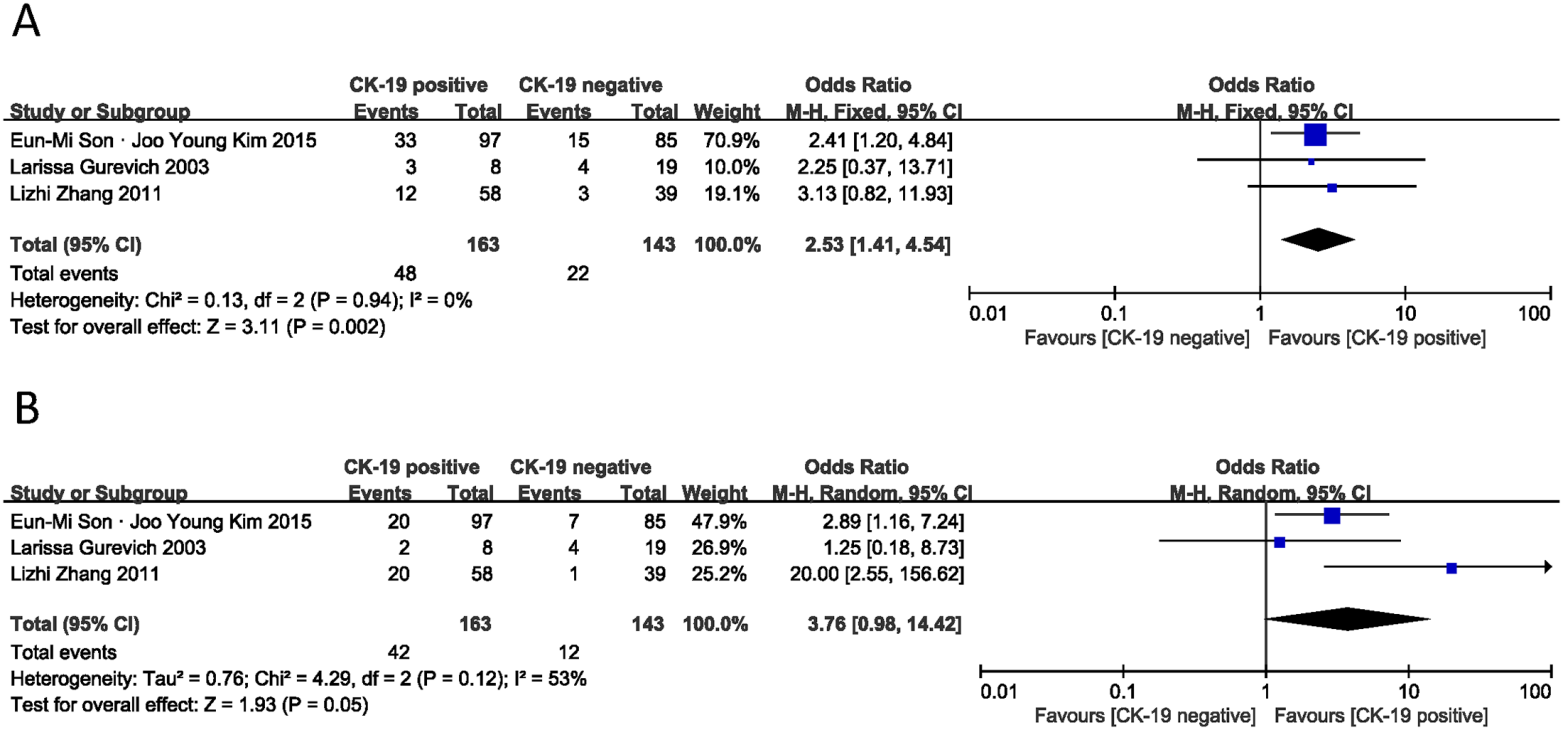
Forest plot displaying the results of the meta-analysis on vascular invasion and perineural invasion.

The two major invasion behaviors of PNET are to vascular and perineural tissues. The vascular invasion result was derived from 3 studies [14, 16, 22]. As shown in Fig 5, the combined OR was 2.53 (95% CI: 1.41–4.54, Z = 3.11, I^2^ = 0%, P = 0.002), meaning that CK-19 expression was associated with vascular invasion. However, CK-19 expression was not significantly correlated with perineural invasion. The pooled OR was 3.76 (n = 3 studies, 95% CI: 0.98–14.42, Z = 1.93, P = 0.05) and statistical heterogeneity was also significant (I^2^ = 53%) (Fig 5).

**Fig 6:**
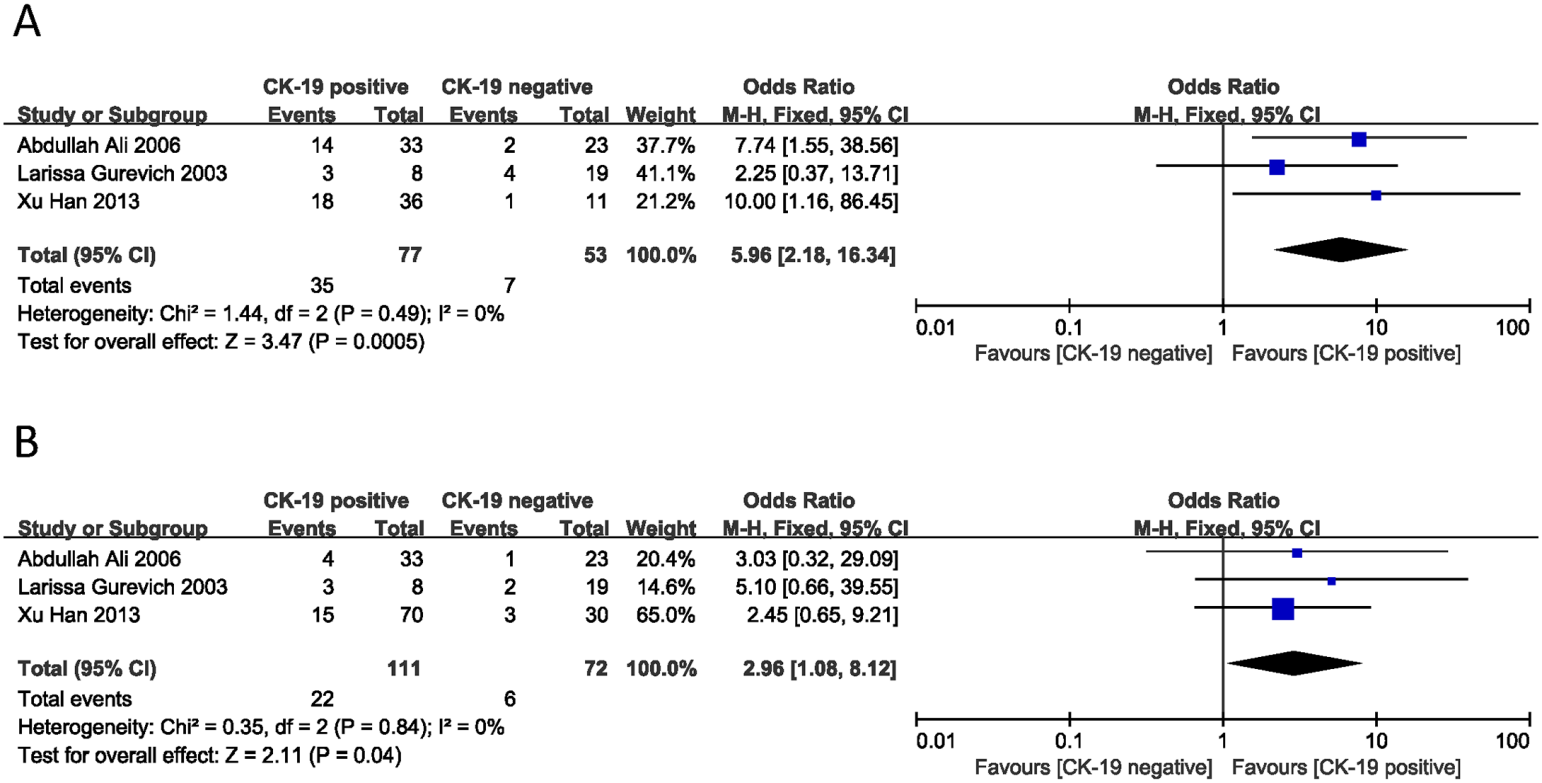
Forest plot displaying the results of the meta-analysis on metastasis.

Metastasis is another aspect for the evaluation of PNET. In this meta-analysis, the correlation between CK-19 expression and lymph node and liver metastasis was described [6, 20, 22]. The combined ORs were 5.96 (95% CI: 2.18–16.34, Z = 3.47, P = 0.0005) for lymph node metastasis and 2.96 (95% CI: 1.08–8.12, Z = 2.11, P = 0.04) for liver metastasis (Fig 6). No statistical heterogeneity was found in either analysis (I^2^ = 0% and 0%). The result showed that PNET with positive CK-19 expression was more likely to have lymph node and liver metastasis than those without CK-19 expression.

### Publication bias

In this meta-analysis, Begg’s test and Egger’s test were used to evaluate potential publication bias. Based on the Begg’s test, no publication bias was found in any study. However, publication bias was found in the studies of 3-year overall survival (P = 0.028) and 5-year overall survival (0.014) in Egger’s test.

## Discussion

PNET is an uncommon tumor, occuring in one to five individuals per 1,000,000 per year [1, 3]. PNET is usually found in adults and rarely in children [23]. The PNET patients are typically 30–60 years. Moreover, men and women are equally affected, but poorly differentiated PNET occurs more frequently in men [5]. PNET originates from the multipotent ductular or acinar stem cells and nonislet cells [24, 25]. Four hereditary syndromes (multiple endocrine neoplasia type 1 (MEN1), von Hippel–Lindau syndrome (VHL), neurofibromatosis type 1 (NF1), and tuberous sclerosis) are associated with PNET [26]. The clinical features depend on whether tumors are functional or non-functional. The functional tumors produce relevant hormone and exhibit a certain endocrine syndrome, while non-functional ones present symptoms, such as abdominal pain, anorexia, nausea, and jaundice, due to the tumor mass, invasion of adjacent tissue or distant metastasis. Currently, radiological, metabolic, serum, and endoscopic strategies are combined to make more precise diagnosis. Surgical resection is curative for localized or early stage PNET. For advanced PNET, systemic treatment or targeted treatment has been implemented, such as somatostatin analogs, agents targeting the vascular endothelial growth factor (VEGF) signaling pathway and the mechanistic target of rapamycin (mTOR). Cytotoxic chemotherapy is another option for patients [27].

Cytokeratins (CK) are a kind of intermediate filament protein. CK-19 is an acidic protein type I, a subgroup of CK. The abnormal expression of CK-19 is associated with different kinds of cancers, including breast cancer, hepatocellular carcinoma, gallbladder cancer andpapillary thyroid carcinoma, by interaction with other biomarkers. CK-19 maintains the function of keratin filament assembly by the phosphorylation of Ser-35 [28]. CK-19 also plays a key role in HCC with the expression of invasion-related/metastasis-related markers (VASP, LAMB1, PDGFRA), biliary marker (CD133, GSTP1, JAG1) and members of microRNA family 200, especially in PDGFRA-LAMININ B1-CK-19 cascade [29, 30]. In lung cancer, CK-19 intracellularly binds to HER2 to promote HER2 activation [31]. Additionally, CK-19 is related to V600E in papillary thyroid carcinoma [32]. CK-19 is mainly expressed in the pancreatic ductal epithelium. During pancreatic morphogenesis, CK-19 is expressed in duct-like pancreatic precursor cells, including pancreatic islet cells between 12 and 16 weeks of fetal development, which will develop into exocrine acinar and islet beta-cell with CK-19 negative expression [10, 11, 33]. Furthermore, PNET with biomarkers of pancreatic precursor cells presents poorly differentiated grade and malignant behavior, while those that do not express biomarkers show better prognoses[6]. Hence, CK-19 can be a key indicator for PNET prognosis.

With the intensive awareness of patients for PNET and improved technology of computer tomography, the detection of PNET is increasing. Although PNET has a relatively good survival rate due to its indolent behavior [34], it is difficult to predict localized behavior, metastasis and recurrence. The prediction of malignant behavior and prognosis has not been successful or efficient using the WHO-2010 classification or the European Neuroendocrine Tumor Society (ENETS) staging system [35, 36]. Therefore, it is urgent to find a new biomarker to evaluate the prognosis of PNET. A large number of studies have focused on CK-19 to evaluate its relationship to the prognosis of PNET. But some debates remain.

Our meta-analysis of the 10 selected studies revealed a total of 856 tumor samples from 854 PNET patients were included, of which, 428 showed positive CK-19 expression. The results indicated that CK-19 expression was significantly associated with 3-year and 5-year overall survival but not 1-year. Compared with ductal adenocarcinoma in the pancreases, PNET patients’ survival rate is apparently longer. Therefore, during the 1^st^ or even 3^rd^ years, it is not very important to evaluate the PNET patients’ survival. This may partially explain why there is no significant correlation between the CK-19 expression and 1-year overall survival. We also performed a meta-regression analysis to detect the effect of confounding factors on the impact of CK-19 on survival outcome. Since most data regarding demographic and clinicopathological features were not available from all original articles, only functional status was examined in the meta-regression analysis. It had no significant effect on the relationship between CK-19 expression and PNET survival outcome. The above results suggest that positive CK-19 expression is an indicator of reduced survival rate of PNET.

For the differentiation grade, WHO-2004 classification system was applied previously [37]. In WHO-2004, PNETs were categorized as “benign behavior”, “uncertain behavior” and “malignant” (including well-differentiated endocrine carcinomas and poorly differentiated endocrine carcinomas) based on the combination of grade, stage and adjunct prognosticators (vascular and perineural invasion). But here, grade and stage of PNET were separated out. Grade was evaluated by WHO-2010 classification by mitotic activity and Ki-67 index [4]. ENETS and American Joint Committee on Cancer (AJCC) were used to assess the stage [38, 39]. In this meta-analysis, results presented that positive CK-19 expression was correlated with malignant tumors in WHO-2004 and grade 3 in WHO-2010, suggesting positive CK-19 expression could predict advanced differentiation grade.

Whether 2 cm belonged to large or small size groups, there was a significant relationship between positive CK-19 expression and large tumor size, indicating a poor prognosis. Moreover, it was found that CK-19 expression was significantly correlated with vascular invasion, lymph node and liver metastasis. That’s to say, PNET with positive CK-19 is more metastatic and adjacently invasive. Similarly, CK-19 plays an important role in the invasion of HCC [29, 30].

However, some limitations should be elaborated. First, heterogeneity does exist in this meta-analysis because of different basic characteristic among the enrolled studies. A random-effects model was used to weaken the unfavorable effect of variation among studies. Second, some relevant data were extracted from the studies indirectly, which could lead to unavoidable bias. For example, some overall survival data is from Kaplan-Meier survival. Third, relevant data from related studies was limited because of insufficient or incompatible statistical methods in these papers. Two studies described distant metastasis or tumor metastasis or metastasis at diagnosis without distinguishing between lymph node and liver metastasis [14, 18]. The data of lymph node metastasis was inconsistent and was excluded in one study [14]. Fourth, different antibodies and definition standards were used to detect CK-19 expression. The unconformity could also result in inevitable heterogeneity. Fifth, subgroup analysis was not applicable because of the relatively small sample size. Finally, only studies published in English were enrolled. Therefore, a potential ethnic demographic bias may exist.

In this meta-analysis, the relation between the CK-19 expression and overall survival (1-year, 3-year and 5-year) and clinicopathological features, such as tumor size, differentiation grade, vascular and perineural invasion, lymph node and liver metastasis, was studied to assess the impact of CK-19 expression on PNET prognosis. We conclude that CK-19 expression is significantly correlated with poor overall survival and is useful for diagnosing clinicopathology. CK-19 can predict the prognosis of PNET patients.

## Supporting information

S1 File PRISMA checklist. (DOC)
