# Supplementary material for: Prognostic significance of cytokeratin 19 expression in pancreatic neuroendocrine tumor: A meta-analysis
Source: PLoS One. 2017 Nov 14;12(11):e0187588. doi: 10.1371/journal.pone.0187588 (PMC5685577; doi:10.1371/journal.pone.0187588)
Supplement: S1 File — (DOC) [file pone.0187588.s001.doc]

| **Section/topic** | **#** | **Checklist item** | **Reported on page #** |
| --- | --- | --- | --- |
| **TITLE** | | |  |
| Title | 1 | Prognostic Significance of Cytokeratin 19 Expression in Pancreatic neuroendocrine tumor: A Meta-Analysis | P1 |
| **ABSTRACT** | | |  |
| Structured summary | 2 | Abstract  Backgrounds: Pancreatic neuroendocrine tumor (PanNET) comprises 1-2% of all pancreatic tumors and its incidence is increasing. Although many studies have investigated the correlation between the cytokeratin 19 (CK-19) and PanNET, the prognostic significance of positive CK-19 expression for PanNET remains inconclusive.  Methods: Eligible studies were retrieved from Pubmed, Elsevier, Embase, Cochrane Library and Web of Science databases. All relevant data was extracted to whether and how postive CK-19 expression impacted the clinicopathological features and the prognosis of PanNET. We utilized a fixed or random effects model to calculate pooled odds ratios (OR) with 95% confidence intervals (CI).  Results: A total of 856 tissue samples from 854 patients in ten studies were enrolled in this meta-analysis. Pooled data indicated that positive CK-19 expression was significantly associated with poor 3- and 5-year overall survival (OS) for PanNET. But there is no significance between positive CK-19 expression and 1-year OS. The combined ORs were 0.45 (n=5 studies, 95% CI: 0.17- 1.22, Z=1.57 , P=0.12) for 1-year OS, 0.34 (n=5 studies, 95% CI: 0.18-0.63, Z=3.45 , P=0.0006) for 3-year OS with no statistical heterogeneity (I2=0% and 45%) and 0.23 (n=5 studies, 95% CI: 0.08- 0.69, Z=2.63 , P=0.008) for 5-year OS with significant statistical heterogeneity (I2=62%). Meanwhile, positive CK-19 expression was also correlated with large tumor size (n=3 studies, OR=2.06, 95% CI: 1.25-3.40, Z=2.82, P=0.005; n=3 studies, OR=2.89, 95% CI: 1.23-6.79, Z=2.43, P=0.01), advanced differentiation grade according to World Health Organization-2010 (WHO-2010) (n=3 studies, OR=3.83, 95% CI: 1.45-10.10, Z=2.71, P=0.007) and WHO-2004 (n=3 studies, OR=4.43, 95% CI: 2.22-8.85, Z=4.22, P<0.0001), vascular invasion (n=3 studies, OR=2.53, 95% CI: 1.41-4.54, Z=3.11, P=0.002), lymph node metastasis (n=3 studies, OR=5.96, 95% CI: 2.18-16.34, Z=3.47, P=0.0005)and liver metastasis (n=3 studies, OR=2.96, 95% CI: 1.08-8.12, Z=2.11, P=0.04).  Conclusions: Positive CK-19 expression can serve as an indicator for the poor prognosis of the patients with PanNET. | P1-2 |
| **INTRODUCTION** | | |  |
| Rationale | 3 | Cytokeratin (CK) or simply keratin, mainly expressed in epithelial cells and skin appendages, is the largest subgroup of intermediate filament proteins. Especially for the pancreas, CK-19 is normally expressed in the exocrine ducts but not in the exocrine acinar and endocrine islet cells. During the pancreatic morphogenesis, duct-like pancreatic precursor cells with CK-19 positive develop into exocrine acinar and islet beta-cell without CK-19. | P2 |
| Objectives | 4 | CK-19 expression was found in PanNET. Lots of studies have been done to research the impaction of CK-19 expression to the prognostic significance of PanNET patients but remain controversial. Therefore, based on the currently available medical literature about the prognosis of the CK-19 expression in PanNET patients, a meta-analysis was performed to investigate whether and how CK-19 expression impacted the prognosis of PanNET in this paper. | P2 |
| **METHODS** | | |  |
| Protocol and registration | 5 | Review Manager (RevMan) software (version 5.2; Cochrane collaboration, http:ims.cochrane.org/revman/download) | P4-5 |
| Eligibility criteria | 6 | All studies included in this meta-analysis had to fulfill the following criteria: (1) patients with distinctive pancreatic neuroendocrine tumor by pathology but without restriction on age or ethnicity; (2) CK-19 expression was measured by immunohistochemistry (IHC); (3) clinical trials or reports were published in English; (4) valuable data was provided directly or could be calculated indirectly; (5) the study with the highest quality assessment was enrolled when trials were performed in the same patient samples. | P5 |
| Information sources | 7 | The Pubmed, Elsevier, Embase, Cochrane Library and Web of Science databases | P1 |
| Search | 8 | Terms of ‘‘Keratin 19’’, ‘‘Cytokeratin 19’’, ‘‘Cytokeratin-19’’ and ‘‘pancreatic endocrine tumor’’, ‘‘pancreatic endocrine neoplasm’’, ‘‘pancreatic neuroendocrine tumor’’, ‘‘pancreatic neuroendocrine neoplasm’’ with all possible combinations were used to filter out all the eligible articles and look through their reference lists for additional available studies. | P3 |
| Study selection | 9 | Abstracts, editorials, letters and expert opinions, conference records or abstracts, book sections, reviews without original data, case reports and studies lack of control groups were excluded. Studies were also excluded if: (1) articles about animals or cell lines; (2) the outcomes or parameters of patients were not clearly reported; (3) conference records or abstracts; (4) no related data required for necessary analysis; (5) overlapping articles. | P3 |
| Data collection process | 10 | Independently, valid data were retrieved from eligible studies by two reviewers (DC and JC). All relevant text, tables and figures were reviewed for data extraction. Any divergence was ironed out by discussion with the third reviewer (RM) for final expectation of consensus. | P3 |
| Data items | 11 | Relevant characteristics were listed as follows: (1) first author’s name; (2) publication date; (3) number of patient population included in this meta-analysis; (4) characteristics of including study population, such as age, gender and clinicopathological features; (5) the disease stage according to WHO-2010 classification and WHO-2004 classification; (6) the methods used to evaluate CK-19 expression; (7) corporations of antibody; (8) percentage of CK-19 expression; (9) whether provided overall survival data (Table 1). | P4 |
| Risk of bias in individual studies | 12 | Chi-square based Q statistical test and I^2^ statistic were used for assessing risk of bias of individual studies at the outcome level. Heterogeneity was significant and unacceptable while I^2^ statistic was greater than 50%. P<0.05 was considered statistically significant. | P5 |
| Summary measures | 13 | Calculated pooled odds ratios (OR) and weighted mean differences (WMD) with 95% confidence intervals (CI) and measured 1/3/5 OS. | P5 |
| Synthesis of results | 14 | Odd ratio (OR), together with 95% confidence interval (CI), was analyzed to estimate whether and how CK-19 expression impacts the prognosis of PanNET. A combined OR<1 suggested a worse survival rate and for clinicopathological features, a combined OR>1 indicated a poor outcome. Heterogeneity among enrolled studies was checked by Chi-square-based Q statistical test. And I^2^ statistic, ranging from 0% to 100%, was also calculated for the inter-study heterogeneity. If a P value for Q-test is less than 0.10 and/or I^2^>50% indicating the presence of heterogeneity in those studies, a random-effects model was used. Otherwise, a fixed-effects model was chosen. The publication bias was evaluated by the funnel plots made by Egger’s test and Begg’s test. If the plots were asymmetrical, the stability of our meta-analysis results need to be assessed by using trim and fill analyses. P<0.05 in Q statistical test was considered statistically significant. | P5 |

| **Section/topic** | **#** | **Checklist item** | **Reported on page #** |
| --- | --- | --- | --- |
| Risk of bias across studies | 15 | (1) heterogeneity brought about by the difference of patient characteristics which was based on to group in all studies; (2) bias about clinical evidence; (3) publication bias; (4) language bias; (5) heterogeneity brought about by the difference of the antibodies; (6) bias led to by other clinical characteristics of patients such as age, sex, different chemotherapies and radiotherapies. | P8 |
| Additional analyses | 16 | Neither sensitivity nor subgroup analyses was used in the meta-analysis considering the availability of present methods and the lack of heterogeneity across studies. |  |
| **RESULTS** | | |  |
| Study selection | 17 | A total of 193 potentially eligible studies were retrieved based on the search strategy given above. 33 articles were excluded that were duplicate studies. Then 88 studies were excluded for the following reasons: case reports, book sections, reviews, animal studies, conference records or abstracts, having no relationship with the topic and having no full text. Finally, after reading the full text, we excluded 8 studies because of lacking of sufficient information about survival or clinicopathological features. Therefore, 10 studies met our inclusion criteria and were used in this meta-analysis (Figure 1). | P5 |

| Study | Year | Countray | Number of Patients | Mean Age | Gender(M/F) | Level of Evidence | WHO grade（2004） | WHO grade(2010) | Clinicopathological Feature | Method | Clone Number of Antibody(Source) | Dilution | Increased CK-19 Expression | Definition Standard | Provided OS Data |
| --- | --- | --- | --- | --- | --- | --- | --- | --- | --- | --- | --- | --- | --- | --- | --- |
| Son et al (14) | 2015 | Korea | 182 | 51.4±13.10 | 81/101 | 6 | NR | G1、G2、G3 | TS, LN, VI, PI | IHC | Cell Marque, Rocklin, CA, USA | 1:100 | 97/182 | >5% | YES |
| Jovenel Cherenfant (15) | 2014 | America | 128 | 55 ± 14 | 71/57 | 6 | NR | G1、G2、G3 | NR | IHC | Biocare, Concord, CA | 1:10 | 82/128 | NR | NO |
| Xu et al (6) | 2013 | China | 100 | NR | NR | 6 | NR | G1、G2、G3 | LN, LM | IHC | Dako, Glostrup, Denmark | 1:300 | 70/100 | >5% | YES |
| Zhang et al (16) | 2011 | America | 97 | 53.9 (54; 22-82) | 51/46 | 6 | NR | NR | VI, PI | IHC | RCK108, DAKO, Carpinteria, CA | 1:20 | 58/97 | >5% | NO |
| Jonkers et al (18) | 2007 | Netherlands | 50 | NR | NR | 4 | B、UB、M | NR | TS | IHC | RCK108, MUbio products BV, Maastricht, The Netherlands | 1:200 | 14/50 | >5% | YES |
| S La Rosa (17) | 2007 | Italy | 136 | NR | NR | 4 | B、UB、WDEC、PDEC | NR | NR | IHC | RCK108, DAKO | 1:100 | 30/136 | >5% | YES |
| Jonkers et al (19) | 2006 | Netherlands | 22 | NR | NR | 4 | B、UB、M | NR | NR | IHC | RCK108, Mubio, Maastricht, the Netherlands | 1:200 | 4/22 | >10% | NO |
| Ali et al (20) | 2006 | Canada | 56 | 49.8 | 26/30 | 6 | NR | NR | TS, LN, LM | IHC | Novocastra, Newcastle, UK | 1:500 | 33/56 | >5% | NO |
| Deshpande et al (21) | 2004 | America | 54 | NR | NR | 4 | NR | NR | NR | IHC | Dako Co., Carpenteria,CA | 1:10 | 28/54 | NR | YES |
| Gurevich et al (22) | 2003 | Russia | 29 | 47 | 9/20 | 5 | NR | NR | TS, LN， VI， PI, LM | IHC | RCK108，Dako (Glostrop, Denmark) | 1:100 | 12/31 | >10% | NO |
| NR:not reported; B:benign; UB:uncertain behavior; M:malignant; WDEC:well-differentiated endocrine carcinomas; PDEC:poorly differentiated endocrine carcinomas; G1:grade 1; G2:grade 2; G3:grade 3; TS:tumor size; VI:vascular invasion; PI:perineural invasion; LN:lymph node metastasis; LM:liver metastasis;IHC:immunohistochemistry; OS:overall survival | | | | | | | | | | | | | | | |

| Risk of bias within studies | 19 | **Figure S2**  **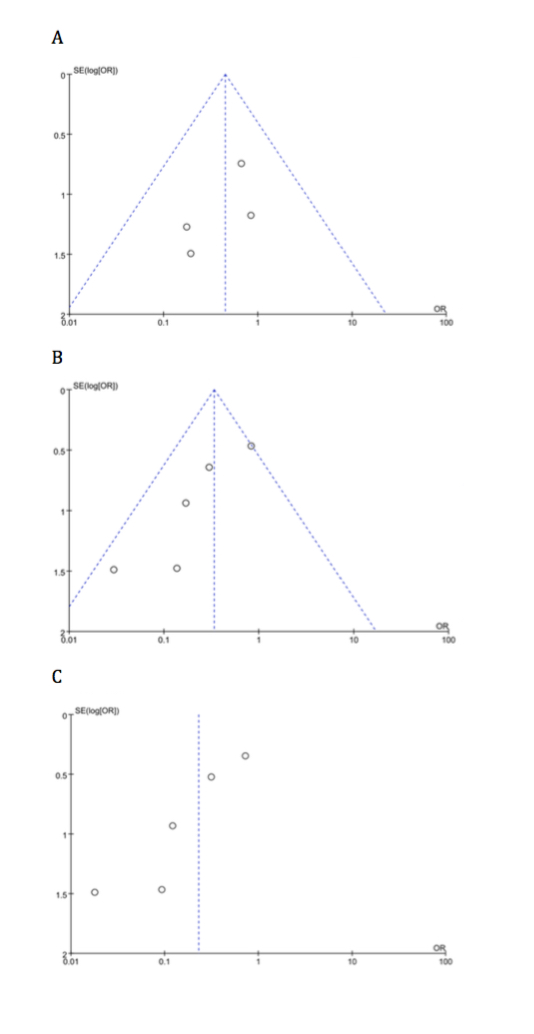**  **Figure S2a**  **Funnel plot to assess publication bias.** Egg’s publication bias plot showed no publication bias for studies regarding *positive* CK-19 expression and 1-year overall survival (OS) in the meta-analysis.  **Figure S2b**  **Funnel plot to assess publication bias.** Egg’s publication bias plot showed the presence of publication bias for studies regarding *positive* CK-19 expression and 3-year OS in the meta-analysis.  **Figure S2c**  **Funnel plot to assess publication bias.** Egg’s publication bias plot showed the presence of publication bias for studies regarding *positive* CK-19 expression and 5-year OS in the meta-analysis.  **Figure S3**  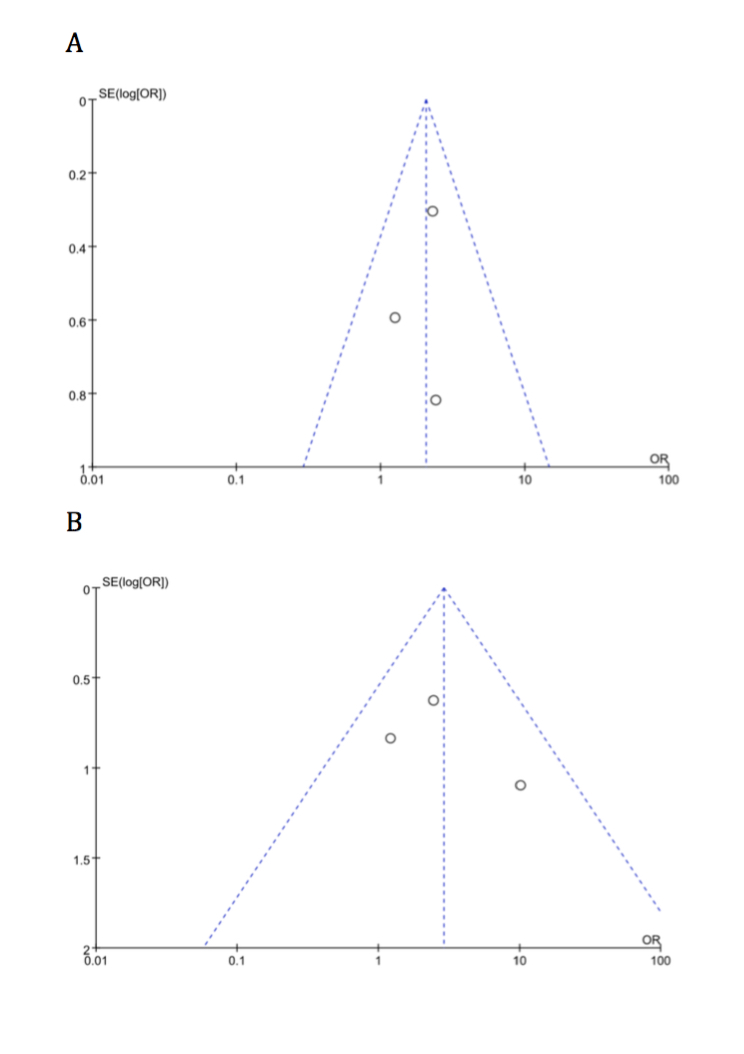  **Figure S3a**  **Funnel plot to assess publication bias.** Egg’s publication bias plot showed no publication bias for studies regarding *positive* CK-19 expression and tumor size one in the meta-analysis.  **Figure S3b**  **Funnel plot to assess publication bias.** Egg’s publication bias plot showed no publication bias for studies regarding *positive* CK-19 expression and tumor size two in the meta-analysis.  **Figure S4**  **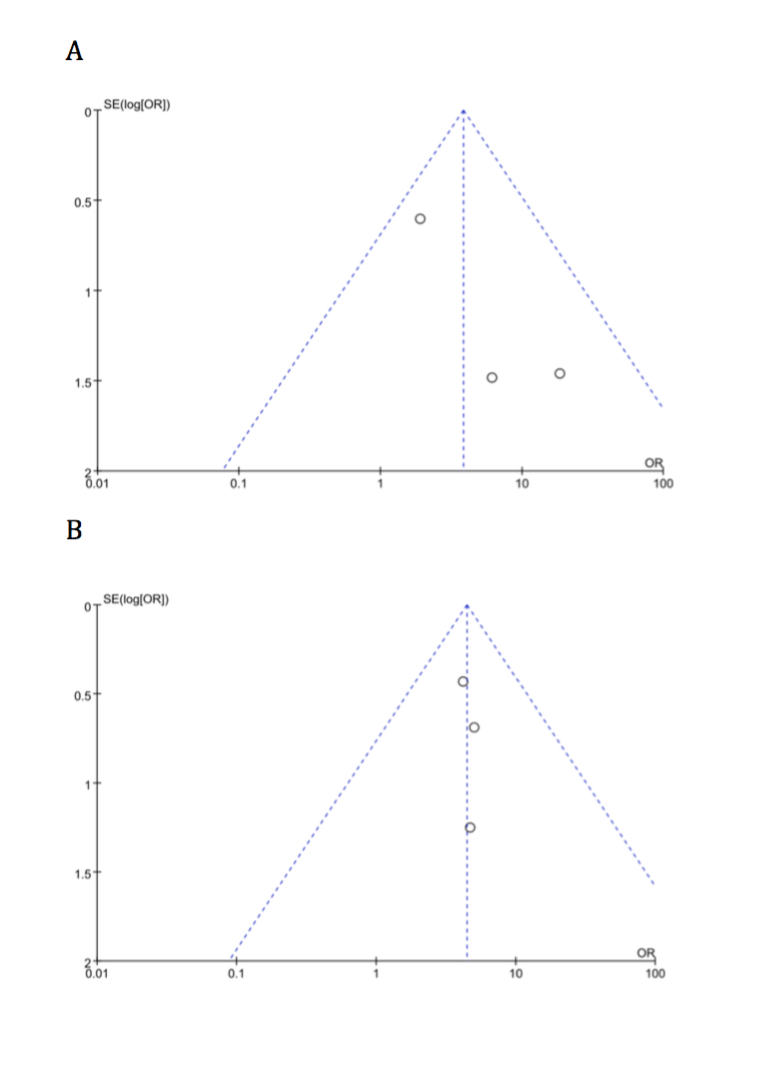**  **Figure S4a**  **Funnel plot to assess publication bias.** Egg’s publication bias plot showed no publication bias for studies regarding *positive* CK-19 expression and WHO-2010 in the meta-analysis.  **Figure S4b**  **Funnel plot to assess publication bias.** Egg’s publication bias plot showed no publication bias for studies regarding *positive* CK-19 expression and WHO-2004 in the meta-analysis.  **Figure S5**  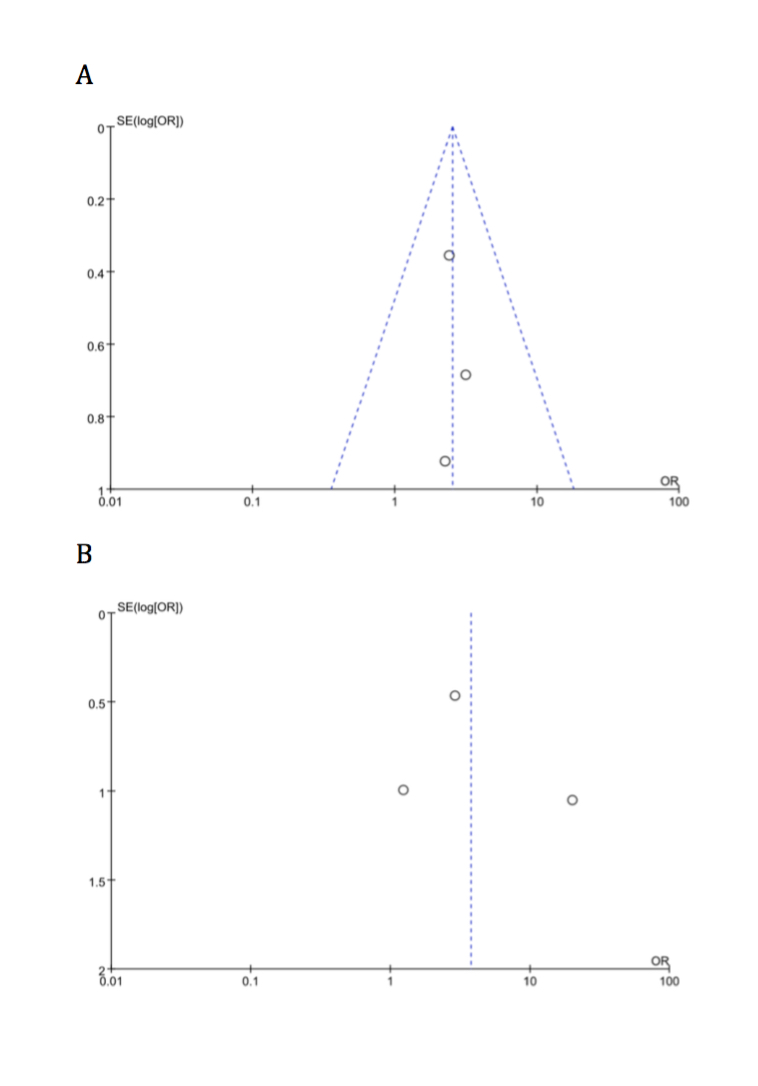  **Figure S5a**  **Funnel plot to assess publication bias.** Egg’s publication bias plot showed no publication bias for studies regarding *positive* CK-19 expression and vascular invasion in the meta-analysis.  **Figure S5b**  **Funnel plot to assess publication bias.** Egg’s publication bias plot showed no publication bias for studies regarding *positive* CK-19 expression and perineural invasion in the meta-analysis.  **Figure S6**  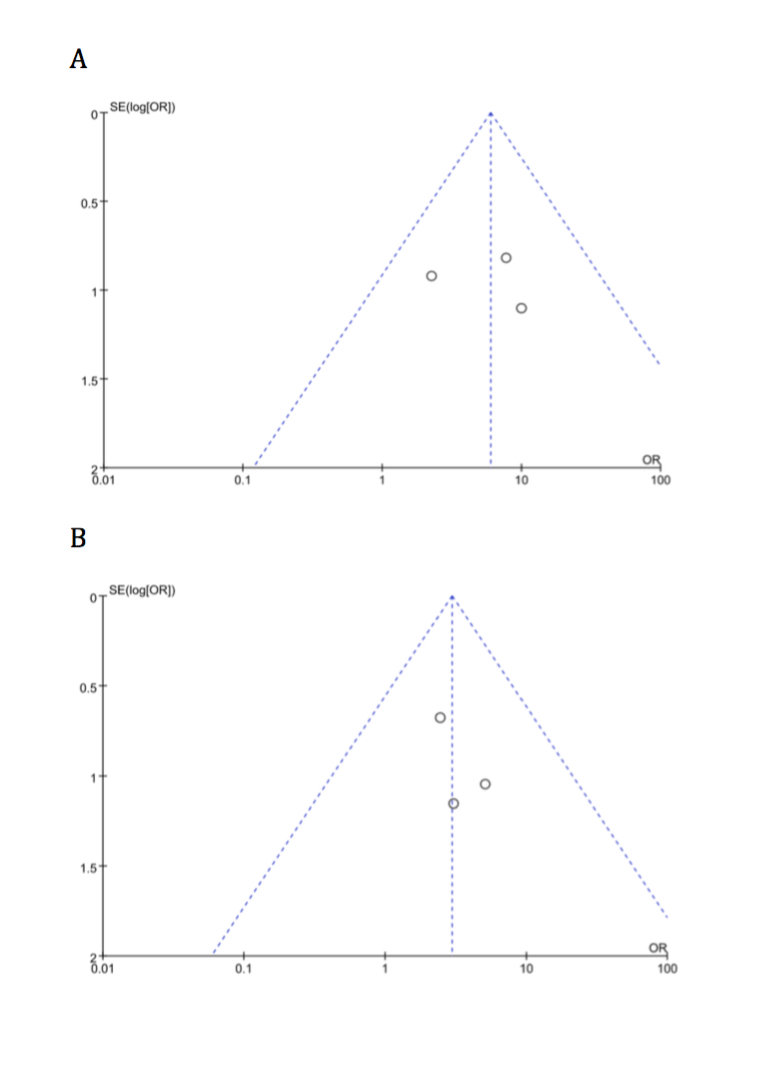  **Figure S6a**  **Funnel plot to assess publication bias.** Egg’s publication bias plot showed no publication bias for studies regarding *positive* CK-19 expression and lymph node metastasis in the meta-analysis.  **Figure S6b**  **Funnel plot to assess publication bias.** Egg’s publication bias plot showed no publication bias for studies regarding *positive* CK-19 expression and liver metastasis in the meta-analysis. |  |
| --- | --- | --- | --- |
| Results of individual studies | 20 | 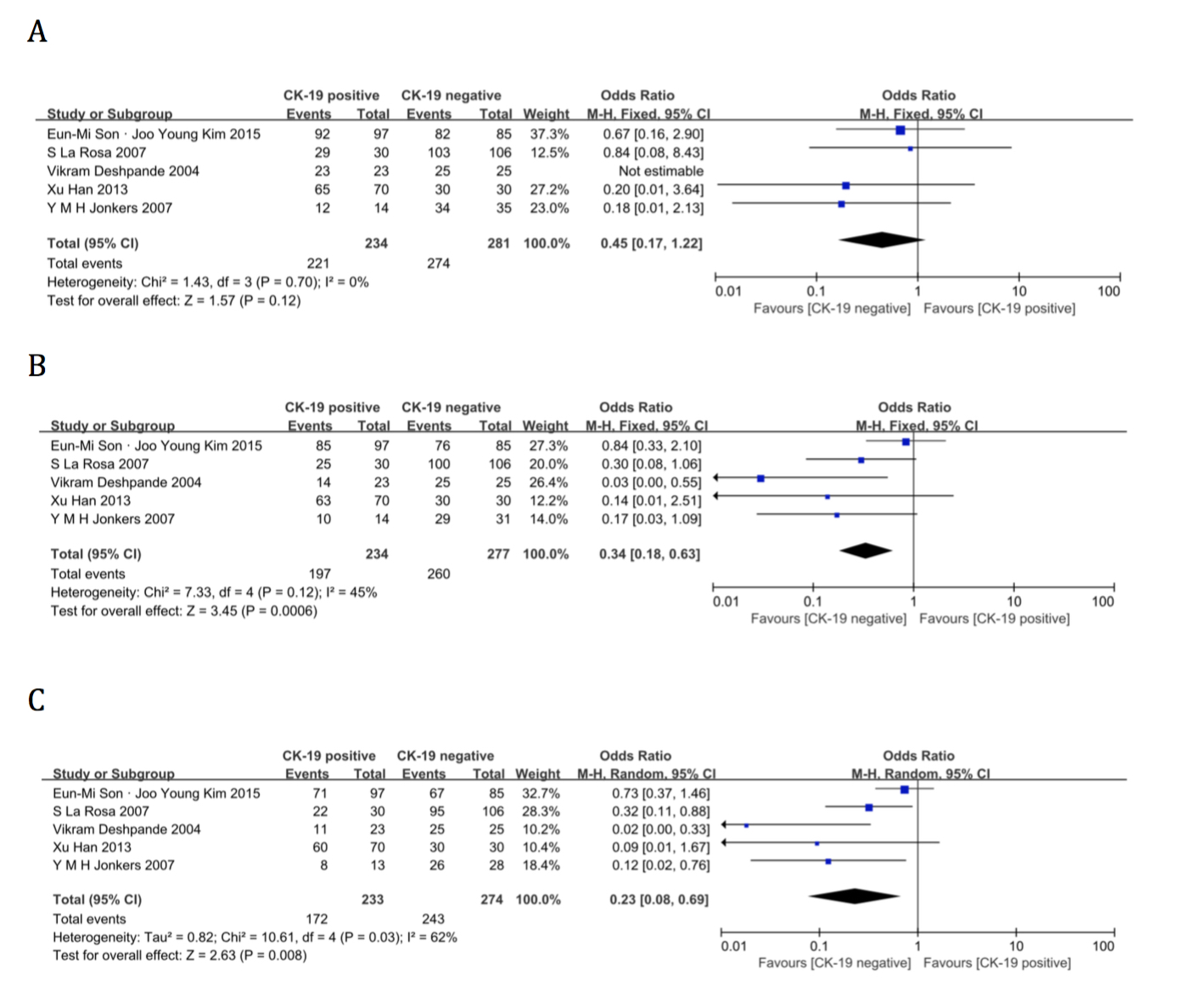  Figure 2 Forest plot displaying the results of the meta-analysis on Overall Survival of 1/3/5 year.  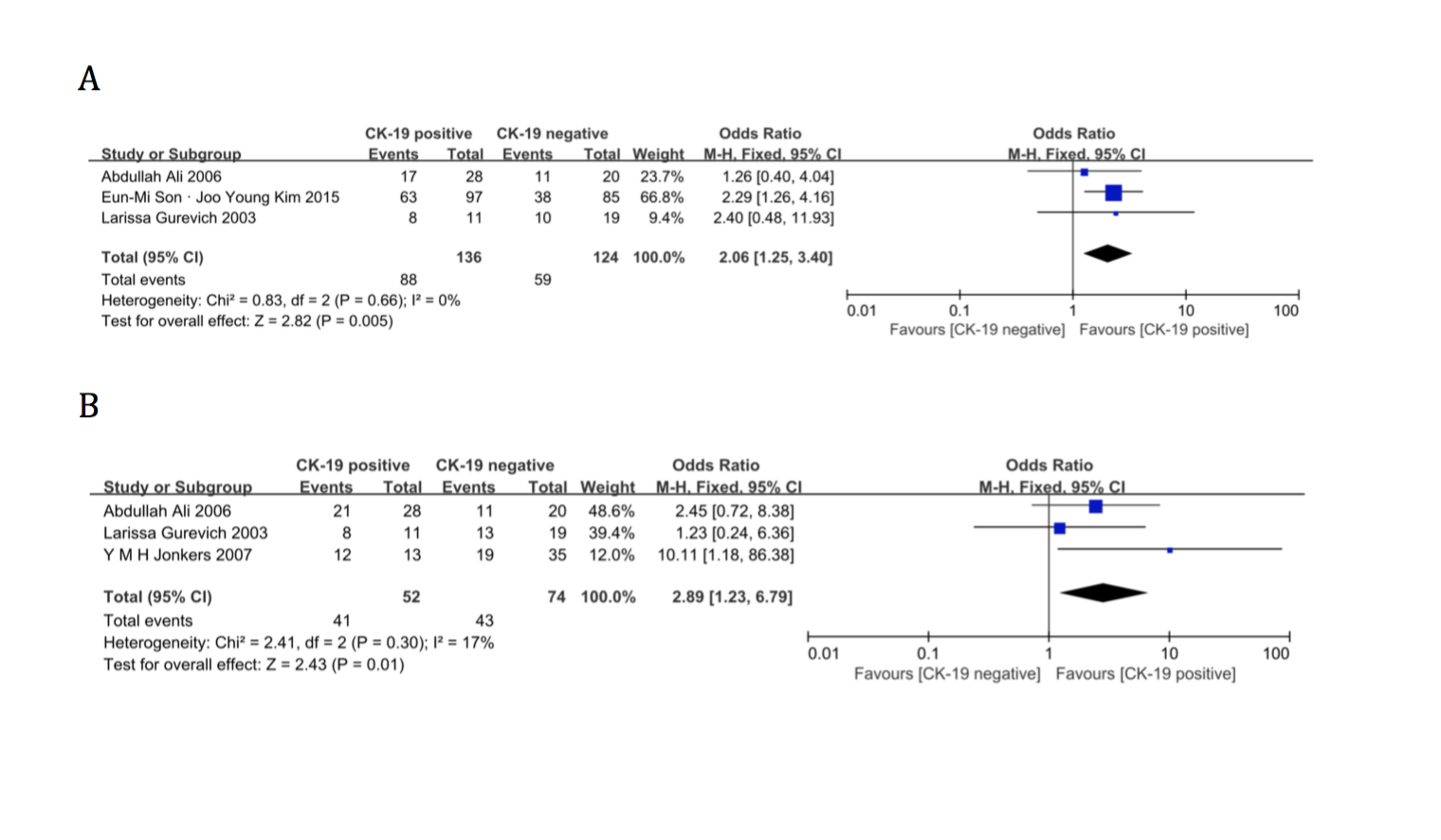  Figure 3 Forest plot displaying the results of the meta-analysis on tumor size.  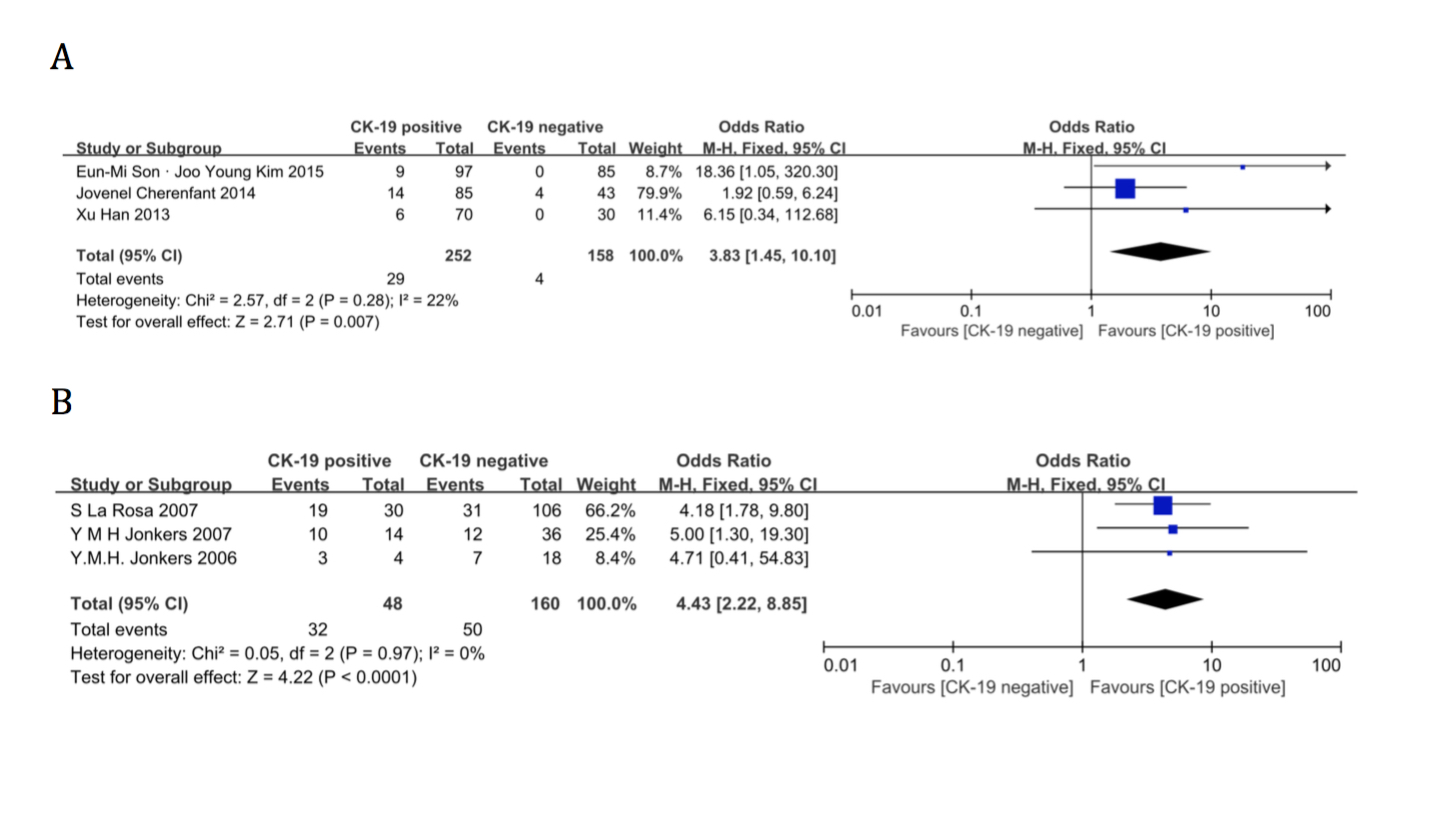  Figure 4 Forest plot displaying the results of the meta-analysis on Differentiation grade of WHO-2010 and WHO-2004.  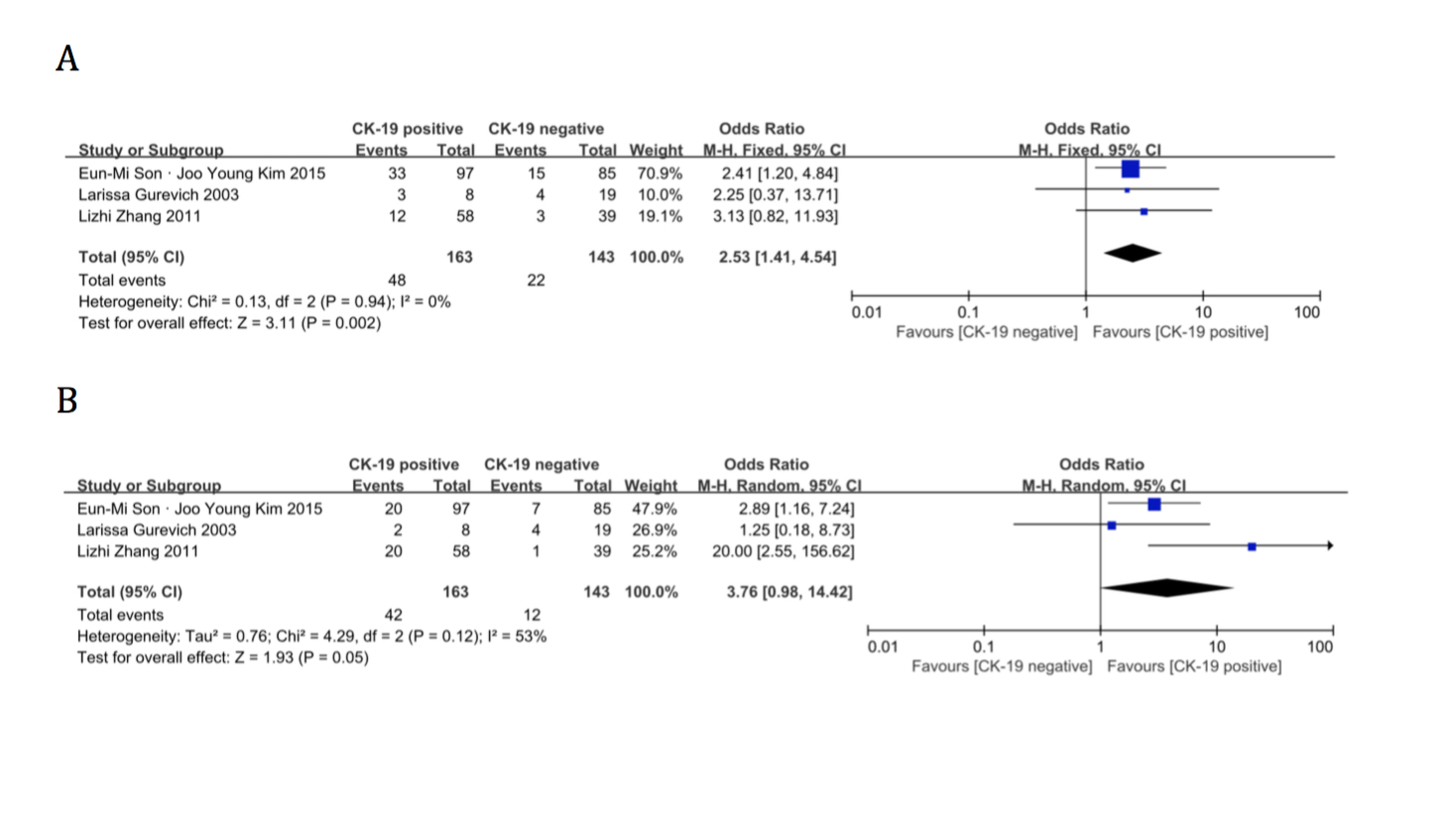  Figure 5 Forest plot displaying the results of the meta-analysis on Vascular invasion and perineural invasion.  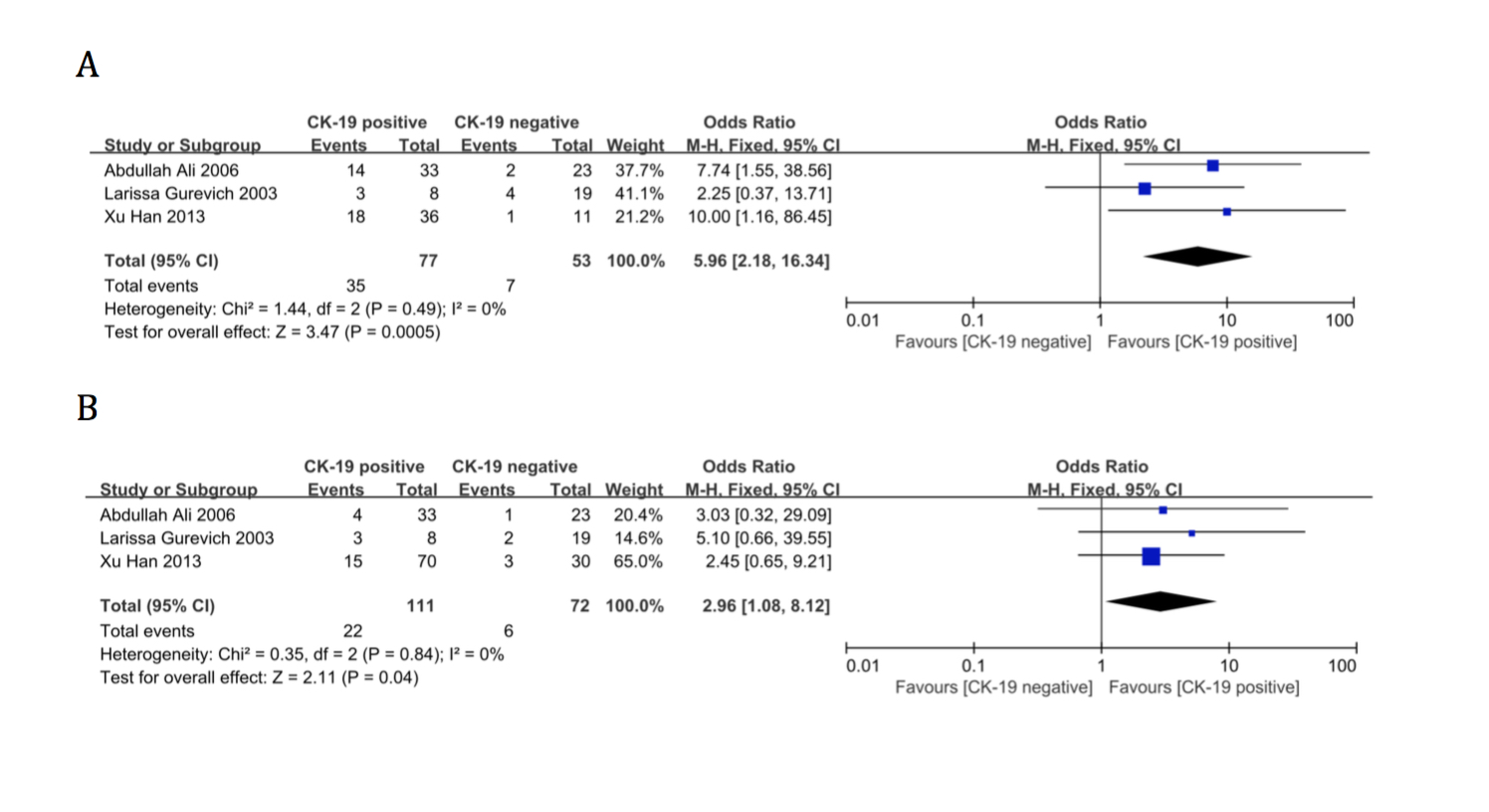  Figure 6 Forest plot displaying the results of the meta-analysis on metastasis. |  |
| Synthesis of results | 21 |  |
| Risk of bias across studies | 22 |  |
| Additional analysis | 23 | None of additional analyses were done. |  |
| **DISCUSSION** | | |  |
| Summary of evidence | 24 | Pooled data indicated that positive CK-19 expression was significantly associated with poor 3- and 5-year overall survival (OS) for PanNET. But there is no significance between positive CK-19 expression and 1-year OS. The combined ORs were 0.45 (n=5 studies, 95% CI: 0.17- 1.22, Z=1.57 , P=0.12) for 1-year OS, 0.34 (n=5 studies, 95% CI: 0.18-0.63, Z=3.45 , P=0.0006) for 3-year OS with no statistical heterogeneity (I^2^=0% and 45%) and 0.23 (n=5 studies, 95% CI: 0.08- 0.69, Z=2.63 , P=0.008) for 5-year OS with significant statistical heterogeneity (I^2^=62%). Meanwhile, positive CK-19 expression was also correlated with large tumor size (n=3 studies, OR=2.06, 95% CI: 1.25-3.40, Z=2.82, P=0.005; n=3 studies, OR=2.89, 95% CI: 1.23-6.79, Z=2.43, P=0.01), advanced differentiation grade according to World Health Organization-2010 (WHO-2010) (n=3 studies, OR=3.83, 95% CI: 1.45-10.10, Z=2.71, P=0.007) and WHO-2004 (n=3 studies, OR=4.43, 95% CI: 2.22-8.85, Z=4.22, P<0.0001), vascular invasion (n=3 studies, OR=2.53, 95% CI: 1.41-4.54, Z=3.11, P=0.002), lymph node metastasis (n=3 studies, OR=5.96, 95% CI: 2.18-16.34, Z=3.47, P=0.0005)and liver metastasis (n=3 studies, OR=2.96, 95% CI: 1.08-8.12, Z=2.11, P=0.04). | P8 |
| Limitations | 25 | Some limitations worthy of further consideration should be elaborated. First, heterogeneity does exist in this meta-analysis because of different basic characteristic in the enrolled studies. A random-effects model was used to weaken the unfavorable effect from the variation among these studies. Second, some relevant data is extracted from the studies indirectly, which could lead to bias in the analysis unavoidably. For example, some OS data is from Kaplan-Meier survival. Third, we could not get enough relevant data from related studies because of the statistical methods in these papers. Two studies described distant metastasis or tumor metastasis or metastasis at diagnosis without giving the precise data of lymph node or liver metastasis. The data of lymph node metastasis was inconsistent and was excluded in one study. Fourth, different antibodies and definition standards were used to detect CK-19 expression in these enrolled studies. The inconformity could also result in inevitable heterogeneity. Fifth, subgroup analysis was not applicable because of the relative small sample size. Finally, only studies published in English were enrolled. Therefore, potential language bias may exist in this analysis. | P10 |
| Conclusions | 26 | In this meta-analysis, the relation between the CK-19 expression and overall survival (1-year OS, 3-year OS and 5-year OS) and clinicopathological features, such as tumor size, differentiation grade, vascular and perineural invasion, lymph node and liver metastasis was studied to assess the impact of CK-19 expression on the prognosis with PanNET patients. In conclusion, positive CK-19 expression was significantly correlated with poor overall survival and clinicopathological situation. That’s to say, positive CK-19 expression have shown the predictive potential for the prognosis of patients with PanNET. | P11 |
| **FUNDING** | | |  |
| Funding | 27 | This work was supported in Zhejiang Provincial Natural Science Foundation of China (Y15H160103). The funder had no role in study design, data collection and analysis, decision to publish, or preparation of the manuscript. | P12 |
